# EHMTI-0328. Comparative evaluation of pregabalin, gabapentin, carbamazepine and topiramat in migraine

**DOI:** 10.1186/1129-2377-15-S1-G7

**Published:** 2014-09-18

**Authors:** A Docu Axelerad, D Docu Axelerad

**Affiliations:** 1 General Medicine, Ovidius University Constanta, Constanta, Romania; 2 Kinetotherapy Department FEFS, Ovidius University Constanta, Constanta, Romania

## Objective

To compare the efficacy and safety of of pregabalin, gabapentin, carbamazepine and topiramat in migraine.

## Research design and methods

In this observational study, 24 patients received pregabalin, gabapentin, carbamazepine and topiramat orally twice daily, each for 6 month with optional dose up titration (divided in four groups, each group has 6 patients). Pain diagnosis of migraine was made according to the second edition of the International Classification of Headache Disorders (ICHD-2) criteria and pain relief was measured by the patient’s global assessment of efficacy, using the Migraine Disability Assessment Questionnaire (MIDAS). Treatment goals include restoring function and improving pain control.

## Results

There was a significant improvement in pain with all treatments compared with their baseline values. There were no significant differences in various other outcome measures between the groups.

## Conclusions

Both carbamazepine and topiramat demonstrated similar efficacy in migraine but pregabalin, gabapentin was less effective on pain.

No conflict of interest.

